# One species, many faces: The underappreciated importance of strain diversity

**DOI:** 10.1371/journal.ppat.1011931

**Published:** 2024-01-25

**Authors:** Jana Nysten, Dimitrios Sofras, Patrick Van Dijck

**Affiliations:** Laboratory of Molecular Cell Biology, Department of Biology, Institute of Botany and Microbiology, KU Leuven, Leuven, Belgium; University of Maryland, Baltimore, UNITED STATES

## Manuscript

Over the past century, extensive efforts have been made to isolate and categorize thousands of strains, resulting in the classification of species as a group of strains that form a coherent genomic cluster [1,2]. Although species delineation is a difficult task and taxonomic methodology can vary depending on taxa and scientists, specific quantitative thresholds have been established to delineate species [3]. Two bacteria are considered to be the same species when their whole-genome average nucleotide identity is at least 95% [4–6]. However, recent research has highlighted significant heterogeneity in genotypes and phenotypes across isolates of the same species despite meeting these genetic similarity thresholds. Take *Escherichia coli* as an example, strains can be host-associated or environmental, a harmless commensal, or even a versatile pathogen [7]. But also within the pathogenic *E*. *coli* strains, there is a lot of variety that hampers risk assessment and strain typing [7]. These strains are divided into intestinal and extraintestinal pathogenic *E*. *coli* which are both further categorized into different pathotypes that have distinct pathogenic traits [7]. The heterogeneity between strains results in differences in genetic circuitry and virulence, affecting the outcome of host infection [8,9]. Thus, exclusively studying reference or wild-type strains may prove inadequate for a comprehensive understanding [1,9,10]. Furthermore, the worrisome global emergence of multidrug-resistant isolates raises the question of the extent to which strain-tailored therapies are needed. In short, this pearl delves into microbial strain diversity, its genetic underpinnings, its impact on host health, its role in antimicrobial resistance, and the challenge of integrating research findings into clinical practice.

## Unveiling the origins and extent of strain diversity

Within-species variability is the result of genomic mutations that arise continuously through errors in DNA replication, DNA repair, recombination mechanisms, and exposure to mutagens like radiation or chemicals [11]. It is important to note that mutations do not automatically translate into functional differences, especially when they occur in intergenic DNA or noncoding regions. For example, up to 98.5% of the human genome can undergo mutations without notable impact [11]. In contrast, microorganisms have more compact genomes with fewer noncoding regions, rendering them more susceptible to significant changes [11]. The extent of within-species variability hinges on factors such as generation time, mutation rate, population size, and the likelihood of inter-species horizontal gene transfer. Subsequently, these changes are shaped by selection and genetic drift, which are modulated by a wide array of biotic (e.g., competition for resources) and abiotic (e.g., antimicrobials) factors [1,12,13].

The extent of within-species variability is contingent upon the particular species under consideration. On one end of the spectrum are monotypic species characterized by populations exhibiting uniform genetic similarities such as *Chlamydia trachomatis* [14]. Typically, these species are specialists with limited host and geographic distributions [15]. Conversely, at the other extreme end, there are polytypic species that are highly diverse generalists, such as *E*. *coli*, showcasing multiple phylogroups [1,16,17]. Thanks to recent advancements in metagenomic sequencing and computational methodologies, researchers have been able to identify strain diversity within species, shedding light on how it can yield functional diversity and affect host outcomes [18].

## The pros and cons of solely relying on reference strains

Microbiologists often use well-characterized laboratory strains known as reference strains or type strains, which are ubiquitous across a research community working with that particular organism. The widespread use of reference or type strains across labs has enhanced reproducibility and allowed researchers to standardize observations and methodologies. While this practice has yielded imperative insights into host–pathogen interactions, recent studies underscore that the substantial genotypic and phenotypic heterogeneity between strains can significantly impact virulence, drug tolerance, and metabolic fitness [8,9,19,20]. For example, in the case of the human fungal pathogen *Candida albicans*, the frequently employed SC5314 reference strain isolated from a bloodstream infection is often used. However, research shows that this strain is rather atypical as it is one of the most filamentous and invasive strains characterized [21]. Consequently, relying solely on a reference strain and generalizing the results can constrain our understanding of a species and its diversity [9].

Recent research has highlighted the importance of strain diversity, revealing that even within a certain host niche, strains can show a rich genetic diversity. Notably, certain *C*. *albicans* isolates were found to lack the most important virulence trait of this species namely hyphae formation which directly influences its cell-damaging capacity and proinflammatory immunity [22,23]. Therefore, carefully considering strain selection becomes pivotal when designing experiments that aim to assess the behavior of pathogens. Isolates are often specialized for a certain host niche and are likely specifically adapted to the immunological and nutritional status of that niche. Such strains might thrive in their original niche but appear defective in other niches or artificial conditions [24]. Acknowledging the high heterogenicity in strains during host–pathogen interactions can bridge the gap between data obtained in controlled laboratory settings and the actual patient outcome [24].

## Strain diversity and its impact on host health

Microbes are often categorized as either pathogenic or commensal [25]. However, it is usually not that straightforward as certain species act as commensals in one host and as pathogens in another, adding complexity to the significance of isolating specific microorganisms in a human host [26,27]. The behavior of a particular species is not solely dependent on the species itself but also on the patient. For example, individuals with compromised immune systems are more susceptible to various diseases. Additionally, genetic, behavioral, cultural, and various other factors may impact the patient’s outcome [28–30]. But to what extent can a different strain influence the patient’s outcome? Within-species variation has been demonstrated to significantly affect virulence in numerous species including *E*. *coli*, *Staphylococcus aureus*, *Mycobacterium tuberculosis*, *C*. *albicans*, *Campylobacter jejuni*, *Listeria monocytogenes*, and *Salmonella enterica* [10,22,31]. The latter bacterial species, a common cause of gastroenteritis, encompasses over 2,500 distinct serotypes that can result in different clinical outcomes [32]. Moreover, even within a serotype, significant differences in virulence-associated genes have been identified [33]. Virulence genes are often situated on distinct pathogenicity islands in the genome or on transmissible genetic elements such as plasmids which facilitate the transmission of genes involved in virulence and pathogenesis. This contributes to the diverse virulence phenotypes exhibited by various isolates [10,32]. Within-species variability can have several effects on the host. Firstly, different isolates inside a host can create antigenic diversity to help evade the host’s immune response [34]. Secondly, benign strains in the gut can protect the host from a secondary infection by training the innate immune response. This phenomenon is referred to as trained immunity or innate immune memory [35,36]. For example, it has been shown that mice that are colonized with benign *C*. *albicans* in the gut are protected from a subsequent intravenously injected lethal *C*. *albicans* challenge [37]. Furthermore, commensal *C*. *albicans* colonization can also confer increased resistance to a variety of systemic infections from other species such as *S*. *aureus*, *Pseudomonas aeruginosa*, and *Aspergillus fumigatus* [35,38]. Interestingly, non-pathogenic microbes can also protect the host against various pathogens [39*]*. Thirdly, studies in *E*. *coli* have revealed that fitness costs associated with resistance acquisition may be diminished through epistasis between resistance genes of different pathogenic isolates [34,40]. Comprehending and considering these variations is essential for customizing effective medical interventions and strategies in public health [18].

## The mosaic of antimicrobial resistance

The use of antimicrobial agents is inextricably intertwined with the emergence of resistance which poses undoubtedly one of the biggest threats to global health [41]. Strain diversity can perplex matters even further by rendering species identification insufficient for the acquisition of meaningful insight towards isolate susceptibility. It has been shown that genetic diversity among bacterial strains cause variation in resistance patterns among different strains of non-O157 Shiga toxin-producing *E*. *coli* against 3 classes of antibiotics [42]. Also, strains of atypical enteropathogenic *E*. *coli* from South Asia and sub-Saharan Africa showed a geographically clustered pattern of resistance, likely due to past usage of antibiotics in those areas [43]. In a large-scale retrospective study including more than 7,000 *E*. *coli* isolates, resistance diversity against 10 antibiotic classes not only differed significantly between strains, but also across different isolation sites, indicating the potential role of niche adaptation in resistance emergence [44]. Similar trends were shown for *Klebsiella pneumoniae* which showed the highest antibiotic resistance, biofilm formation, and beta-lactamase production in strains isolated from the urine compared to those isolated from blood or the respiratory tract [45]. Furthermore, exogenous DNA has been shown to lead to differences in antimicrobial resistance in group A *Streptococcus* isolates [46]. It is also interesting to consider the situation of one host carrying multiple strains. Caballero and colleagues [47] showed that in patients with multiple strains of *Pseudomonas aeruginosa*, resistance developed rapidly, whereas in those with single strains, resistance developed sporadically. Interestingly, resistance may be lost in patients with mixed populations due to fitness trade-offs in the absence of treatment [47]. Shifting the focus to fungi, intraspecies diversity also translates into differential isolate susceptibility, although in a less pronounced manner than in bacteria. In the case of *Cryptococcus neoformans*, the lead cause of deaths for fungal infections, the susceptibility profiles of approximately 300 clinical isolates from the Netherlands showed variation especially for flucytosine and fluconazole [48]. Fluconazole susceptibility also differed for distinct *Candida glabrata* strains isolated from the same patient, while the resistant isolates demonstrated impaired growth compared to the susceptible ones [49]. Differential azole and echinocandin susceptibility was also observed in *C*. *albicans*, the most frequent cause of candidiasis [50]. The emerging fungus *Candida auris* showcases the possibilities of resistance discrepancies due to strain diversity. *C*. *auris* isolates fall into 5 genetically and geographically distinct clades [51,52]. Resistance to fluconazole is common among strains of Clades I and III, whereas amphotericin B resistance is frequent solely for Clade I, and echinocandin resistance is often found within Clade IV isolates [53–55]. Overall, strain diversity is likely crucial in shaping antimicrobial resistance patterns. Understanding this impact is vital for the development of effective strategies to combat it, potentially through targeted interventions and therapies.

## Bridging the gap between research and clinical reality

Finally, it is crucial to critically address the balance between the feasibility and the usefulness of implementing what strain diversity has taught us into everyday clinical practices. As discussed earlier, for research purposes, scientists are advised to select a broad panel of strains to make robust claims, ensuring the diversity within that set adequately represents the diversity within the target microbial species. However, the scenario in clinical settings often demands swift decisions, with treatments often initiated based on medical data, personal preferences, and emotions, even before species identification [56]. The importance of strain diversity in clinical outcomes is unclear as for example in *M*. *tuberculosis* [57], although Gagneux and colleagues have proposed the existence of geographical human–pathogen incompatibility for this pathogen [58]. In certain cases, different strains of the same species may exhibit varying levels of antimicrobial resistance, indicating a need for tailored treatment. Among fungal infections for example, invasive candidiasis is often treated firstly with an echinocandin, and if unsuccessful, a switch to an azole or amphotericin B is made [59]. Noteworthily, treatment with a combination of drug classes would intuitively seem like a good alternative to monotherapy to combat strain-specific resistance, but one has to account for the possible adverse effects and antagonism between drugs [60], as well as the associated higher cost of this approach which is a major hurdle for low-income countries [60,61]. For species with differential susceptibilities between strains, like *C*. *auris*, clade identification prior to the treatment onset, could provide vital information for selecting the first-line therapeutic agent. Cost- and time-efficient clade identification techniques, e.g., PCR-based, have been developed and could be implemented in clinics worldwide [62,63]. However, such in-depth characterization is often beyond the scope and the guidelines of clinicians, particularly for non-specialized infectiologists. Unless future research reveals a necessity for distinct treatments for specific microbial isolates, the delicate balance between strain diversity and practical clinical decision-making will remain a subject of ongoing discussion and adaptation.
